# Focused Review: Cytotoxic and Antioxidant Potentials of Mangrove-Derived *Streptomyces*

**DOI:** 10.3389/fmicb.2017.02065

**Published:** 2017-11-01

**Authors:** Hooi-Leng Ser, Loh Teng-Hern Tan, Jodi Woan-Fei Law, Kok-Gan Chan, Acharaporn Duangjai, Surasak Saokaew, Priyia Pusparajah, Nurul-Syakima Ab Mutalib, Tahir Mehmood Khan, Bey-Hing Goh, Learn-Han Lee

**Affiliations:** ^1^Novel Bacteria and Drug Discovery Research Group, School of Pharmacy, Monash University Malaysia, Bandar Sunway, Malaysia; ^2^Biomedical Research Laboratory, Jeffrey Cheah School of Medicine and Health Sciences, Monash University Malaysia, Bandar Sunway, Malaysia; ^3^Biofunctional Molecule Exploratory Research Group, School of Pharmacy, Monash University Malaysia, Bandar Sunway, Malaysia; ^4^Division of Genetics and Molecular Biology, Faculty of Science, Institute of Biological Sciences, University of Malaya, Kuala Lumpur, Malaysia; ^5^Vice Chancellor Office, Jiangsu University, Zhenjiang, China; ^6^Division of Physiology, School of Medical Sciences, University of Phayao, Phayao, Thailand; ^7^Center of Health Outcomes Research and Therapeutic Safety, School of Pharmaceutical Sciences, University of Phayao, Phayao, Thailand; ^8^Pharmaceutical Outcomes Research Center, Faculty of Pharmaceutical Sciences, Naresuan University, Phitsanulok, Thailand; ^9^UKM Medical Molecular Biology Institute, UKM Medical Centre, Universiti Kebangsaan Malaysia, Kuala Lumpur, Malaysia; ^10^Department of Pharmacy, Absyn University Peshawar, Peshawar, Pakistan; ^11^Asian Centre for Evidence Synthesis in Population, Implementation and Clinical Outcomes, Health and Well-Being Cluster, Global Asia in the 21st Century Platform, Monash University Malaysia, Bandar Sunway, Malaysia

**Keywords:** *Streptomyces*, cytotoxic, antioxidant, mangrove, bioactive

## Abstract

Human life expectancy is rapidly increasing with an associated increasing burden of chronic diseases, such as neurodegenerative diseases and cancer. However, there is limited progress in finding effective treatment for these conditions. For this reason, members of the genus *Streptomyces* have been explored extensively over the past decades as these filamentous bacteria are highly efficient in producing bioactive compounds with human health benefits. Being ubiquitous in nature, streptomycetes can be found in both terrestrial and marine environments. Previously, two *Streptomyces* strains (MUSC 137^T^ and MUM 256) isolated from mangrove sediments in Peninsular Malaysia demonstrated potent antioxidant and cytotoxic activities against several human cancer cell lines on bioactivity screening. These results illustrate the importance of streptomycetes from underexplored regions aside from the terrestrial ecosystem. Here we provide the insights and significance of *Streptomyces* species in the search of anticancer and/or chemopreventive agents and highlight the impact of next generation sequencing on drug discovery from the *Streptomyces* arsenal.

## Introduction

Among primates, humans have the longest life expectancy. With the advancement in medical technologies and improved hygiene conditions, the global life expectancy of a human at birth has increased from 66.4 years in 2000 to 71.4 years in 2015 (Christensen et al., 2009; World Health Organization, 2016). However, there is still much room for improvement in the management of chronic diseases to reduce mortality rate and further extend life expectancy in humans. As part of the aging process, the capacity of the human body to deal with external changes and/or environmental stresses reduces, which eventually leads to development of chronic diseases, such as neurodegenerative diseases (e.g., Parkinson's disease and Alzheimer's disease), cardiovascular diseases and also cancer (Gemma et al., 2007; Abdollahi et al., 2014; Fischer and Maier, 2015; Leszek et al., 2016). One of the main causes that contributes to the development of these chronic diseases is cumulative accumulation of free radicals over time which ultimately overcomes the antioxidant defenses of the host, causing various deleterious effects on cell components and connective tissues. Free radicals are highly reactive molecules with an odd number of electrons in their atomic or molecular orbitals; these harmful radicals can damage cellular lipids, proteins, or DNA, hindering their normal function (Hussain et al., 2003; Valko et al., 2007). Oxidative damage in DNA causes base modifications, single- and double-strand breaks, and the formation of apurinic/apyrimidinic lesions; occurrence of these unwanted DNA modifications which are toxic and/or mutagenic have been implicated in the etiology of various human cancers (Valko et al., 2006). Preventing the accumulation of these reactive free radicals, for instance via high intake of antioxidants has been associated with reduced risk of developing chronic diseases and cancer (Griffiths et al., 2016; Prasad, 2016). Apart from modifying proteins and causing cell damage, free radicals can interfere with the expression of a number of genes and signal transduction pathways, including stimulation of cell proliferation pathways and inhibition of cell death signaling pathways which are commonly seen in various human cancers (Valko et al., 2006).

In the search for novel treatments, researchers have been exploiting bioactive compounds from natural resources to prevent and manage these chronic diseases. Microorganisms have had actually great positive impact on human health and represent an attractive resource for novel drug discovery, as these tiny living organisms are capable of synthesizing structurally-diverse substances with various bioactivities (Demain, 1999; Newman et al., 2000; Berdy, 2005; Demain and Sanchez, 2009). These microbial-derived natural products may be used directly as effective drug(s) or serve as drug lead compounds that could be further modified and developed for higher efficacy. Within the domain Bacteria, streptomycetes are among the most complex bacteria with a life cycle that are more reminiscent of filamentous fungi than of other bacteria. Nonetheless, these filamentous bacteria produce a great variety of secondary metabolites of medical and agricultural interest, including antivirals, antibacterials, antifungals, anticancer, antioxidants and neuroprotective compounds (Ara et al., 2012; Lee et al., 2014a,b; Manivasagan et al., 2014; Azman et al., 2015; Ser et al., 2015b,a, 2016c; Tan et al., 2015; Law et al., 2017). To emphasize this point, approximately 70% of the antibiotics used in medicine were originally derived from actinobacteria (Subramani and Aalbersberg, 2012), of which over 10,000 of these drugs were derived from genus *Streptomyces* (Berdy, 2005; de Lima Procópio et al., 2012). For this reason, continuous efforts have been made to harness the full potential of streptomycetes, specifically in the field of drug discovery with hopes of isolating novel compounds with high efficacy and potency.

## What is *Streptomyces*?

Actinobacteria was once referred as “ray fungi” or Strahlenpilze by Lieske (1921) as this group of Gram-positive bacteria was thought to be the “intermediary” between the fungi and bacteria with a complicated taxonomy. Some growth characteristics of actinobacteria initially confused taxonomists as their colony morphology on agar somewhat resembles the mycelium of fungi with radial growth and most of them give off an earthy-musty odor (Marshall and Alexander, 1960). On the other hand, these filamentous bacteria showed cell wall features that were more similar to other bacteria than fungi, as they consisted of peptidoglycan and teichoic acids with the absence of cellulose and chitin (which can be commonly detected in true fungi). In the face of these inexplicable results, new criteria were introduced to differentiate these bacteria from fungi aside from cellular morphology. These included: (a) assessment of genetic similarity between pools of DNA sequences, (b) measurement of genomic guanine-cytosine (G+C) content, (c) analysis of the cell wall and membrane composition. In bacterial systematics, none of these suggested methods sufficiently describe a microbe when taken individually. However, these pieces of information collectively provide a solid taxonomic basis along with morphological and growth characteristics. These efforts in improving bacterial systematics then allowed re-classification of many actinobacterial lineages beginning in 1967, which included one of the prominent secondary metabolites producers–the genera *Streptomyces* (Williams et al., 1983).

As the largest genus of *Actinobacteria*, members of *Streptomyces* genus have been studied extensively over the past decades. The genus *Streptomyces* was initially proposed by Waksman and Henrici in 1943, a couple of years after the discovery of actinomycin from *Actinomyces antibioticus* (now *Streptomyces antibioticus*). In reality, *S. antibioticus* was first discovered from soil in 1940 by Waksman and Woodruff (1941). This bacterium produced an active compound with specific bacteriostatic and bactericidal properties which was then designated as actinomycin (Waksman and Woodruff, 1940a,b). Apart from inhibiting the growth of some pathogenic bacteria and fungi (bacteriostatic), actinomycin or dactinomycin was the first antibiotic shown to possess anticancer activity; the drug was approved by United States Food and Drug Administrative (FDA) in 1964 under the trade name Cosmegen (Waksman and Woodruff, 1940b; Pugh et al., 1956). This valuable drug is currently listed in the 19th WHO Model List of Essential Medicines (April 2015) as a therapeutic agent against gestational trophoblastic neoplasia, rhabdomyosarcoma and also Wilms tumor. On top of the discovery of actinomycin, Professor Waksman and his team also isolated several new antibiotics from *Streptomyces*, including streptothricin, streptomycin, grisein, fradicin (Waksman et al., 1946; Reynolds et al., 1947; Swart et al., 1950). His passion in actinomycetes research, particularly toward the members of *Streptomyces* subsequently led to the award of the Nobel Prize in Physiology or Medicine in 1952 for the discovery of streptomycin from *Streptomyces griseus*, which was the first effective treatment against the causative agent of the great white plague, *Mycobacterium tuberculosis* (Sakula, 1988).

**Figure 1:**
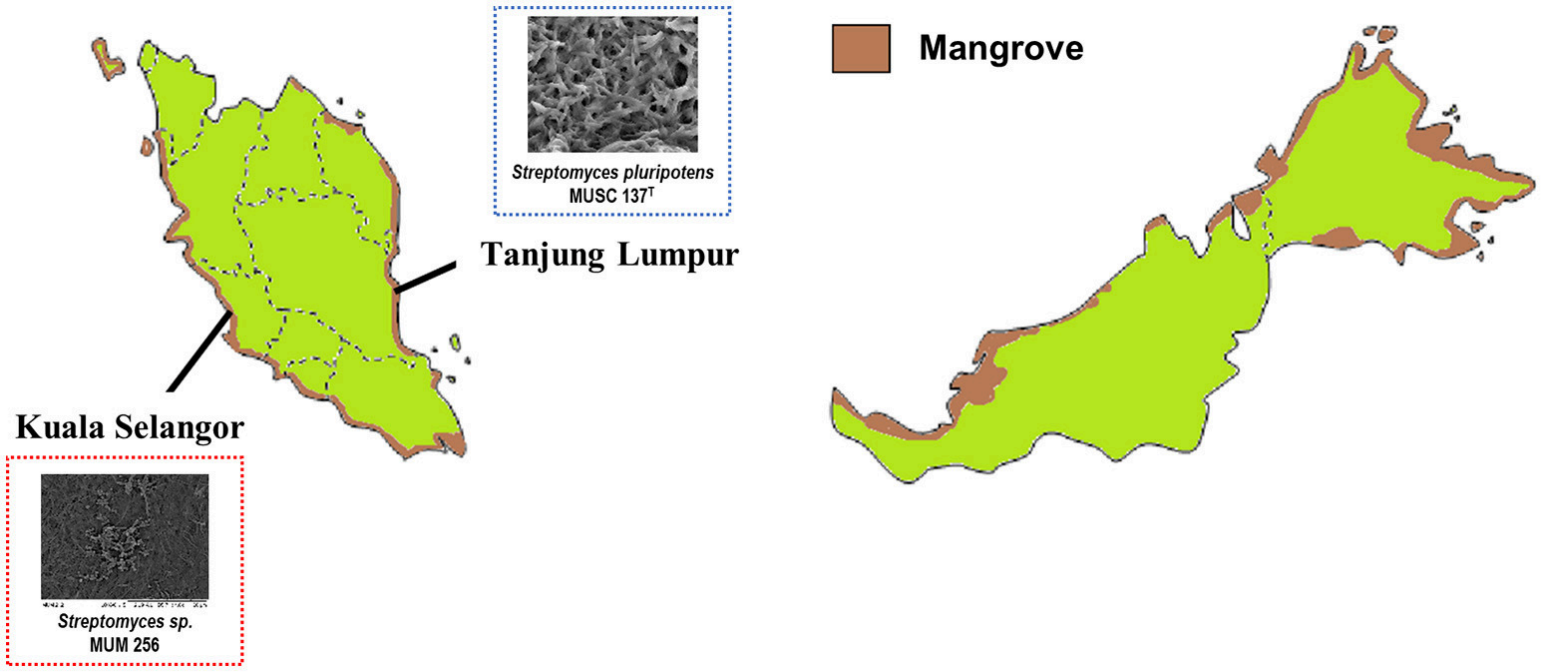
Mangrove distribution in Malaysia (Adapted from ITTO, 2014). The total mangrove coverage of Malaysia was found to be 7,100 km^2^, which equals to 3.7% of total global coverage of mangrove forest. *Streptomyces pluripotens* MUSC 137^T^ was initially isolated from mangrove forest in the east coast of Peninsular Malaysia, while *Streptomyces* sp. MUM 256 was derived from mangrove forest in the west coast of Peninsular Malaysia.

Even after so many years, the search of bioactive compounds from actinomycetes remains a research “hot spot.” Since the isolation of actinomycin as an anticancer agent, many anticancer compounds have been isolated from *Streptomyces* species, including anthracyclines, bleomycin, and mitosanes. For instance, doxorubicin (which belongs to the anthracycline class) was discovered from a soil-derived *Streptomyces peucetius* in 1957 (Grein et al., 1963; Malla et al., 2010). Up to this date, doxorubicin is still in use as chemotherapy against various lymphomas and breast cancer (World Health Organization, 2015). The remarkable findings from these streptomycetes have prompted researchers to take a step further by isolating these microorganisms from other underexplored, extreme and/or special environments, such as coral reef, hot springs and mangrove sediments. With reference to mangrove, Asia is the ideal location for this research as it has the largest coverage of **mangrove forests**, contributing 42% of the global total (Giri et al., 2011; ITTO, 2014). In Southeast Asia alone, Indonesia is home to 22.6% of global mangrove with 3,112,989 hectars of mangrove forest, followed by Malaysia with 505,386 hectars (3.7% of global total; Figure 1). This is significant as mangrove sediments serve as a “mixing zone” between terrestrial and marine habitats and hosts a unique bacterial community (Wang et al., 2012). Compared to marine sediment, several bacterial lineages have been shown to be enriched in mangrove sediments, including *Actinomycetales, Bacteroidetes, Chloroflexi, Firmicutes, Epsilonproteobacteria, Rhodobacterales* (an order within *Alphaproteobacteria*), and *Ectothiorhodospiraceae* (a family within *Gammaproteobacteria*). Previous studies have also claimed that the high abundance of primary producers in mangrove sediments results in the sediment being enriched with high nutrient levels, supporting growth and encouraging species richness. On top of that, numerous studies have also suggested that the stress on the organisms due to the rapid fluctuations in environmental factors in the mangrove environment, such as temperature, salinity and oxygen availability could also promote emergence of novel species by encouraging metabolic pathway adaptations and production of valuable metabolites (Ball, 1998; Ikenaga et al., 2010; Liu et al., 2015). In keeping with these ideas, various actinobacteria have been isolated from mangrove sediments in Malaysia, particularly *Streptomyces* species; the findings from this work has highlighted the important role of exploring the mangrove in bioprospecting.

KEY CONCEPT 1 **Mangrove forests**

As an intertidal region between the land and the sea, mangrove forests constantly experiences fluctuations in temperature, salinity and oxygen availability; these factors are suggested to promote the emergence of novel species along with metabolic pathway adaptations and production of valuable metabolites.

## The life cycle of *Streptomyces* and production of bioactive compounds

**Figure 2:**
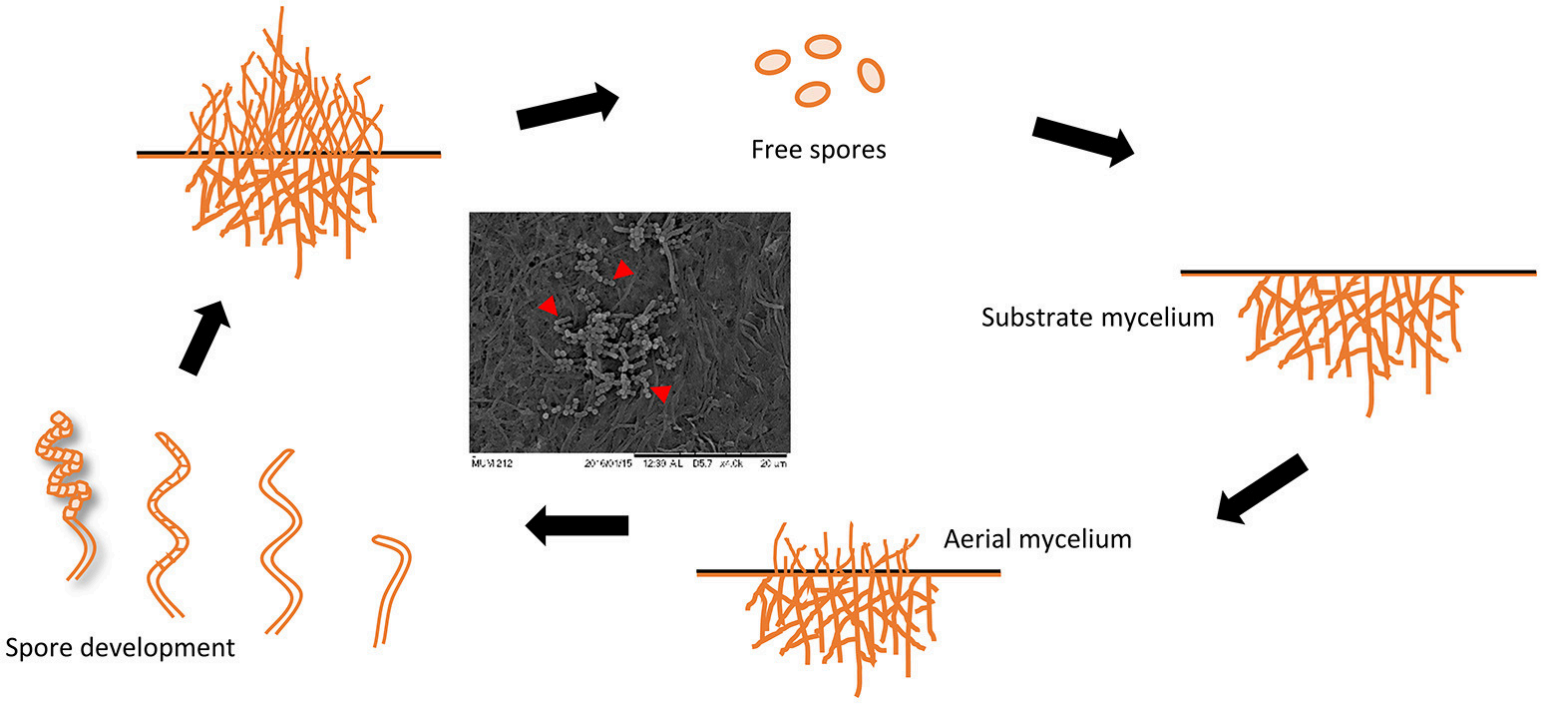
Life cycle of *Streptomyces*. Scanning electron micrograph is showing mycelium of *Streptomyces* sp. MUM 256 and its spores as indicated by red arrows (Adapted from Angert, 2005).

Ironically, the **complicated developmental life cycle of streptomycetes** is one of the reasons that allows them to persist and survive in various environments. The developmental stage of streptomycetes begins with spore germination and outgrowth of substrate (vegetative) mycelium which grow radially with frequent branching and subsequently form a young colony (Figure 2; Wildermuth, 1970; Flärdh and Buttner, 2009; McCormick and Flärdh, 2012; Seipke et al., 2012; Li et al., 2016). As the environment becomes unfavorable for growth (e.g., depletion of nutrition), morphological differentiation will then take place and streptomycetes initiate aerial (reproductive) mycelium formation, undergoing cell division to form spores. The difference in aerial and substrate mycelium color have been one of the principal criterion in characterization and identification of streptomycetes (Pridham, 1965). For example, *Streptomyces pluripotens* MUSC 137^T^ and *Streptomyces* sp. MUM 256 have been cultivated on various agar following the recommendation of International *Streptomyces* Project (ISP; Shirling and Gottlieb, 1966); mixed color of aerial and substrate mycelium have been recorded when comparing across different agars (See Supplementary Table 1). In point of fact, this observation could be essentially similar to what Professor Waksman and his team observed with *S. griseus* way back in 1948 (Waksman et al., 1948); the differential ability of these strains in utilizing certain nutrients could affect their growth on different agar, eventually resulting in the production of different bioactive compounds. With the improvement in phenotypic characterization techniques, researchers can perform high-throughput phenotypic testing in specialized, customizable 96-well plates using newer technologies, such as Biolog OmniLog incubator/readers (Bochner et al., 2001). Compared to the traditional cultivation approach involving growing the strains on different agar, this technology is less labor-intensive and able to generate quantitative phenotypic data by monitoring cell respiration with a tetrazolium dye (Bochner et al., 2001; Borglin et al., 2012). Furthermore, the quantitative readings generated from this technology allow further kinetic studies on utilization of different substrates, which would facilitate the media optimization process to maximize yield of the product of interest.

KEY CONCEPT 2 **Complicated developmental life cycle of streptomycetes**

The unique life cycle of *Streptomyces* sp. allows these filamentous bacteria to be ubiquitous in nature, along with the production of bioactive compounds that promotes their survivability.

The process of media optimization is rarely as easy as it sounds, particularly when it comes to maximizing production of the product of interest by any living microorganisms. As noted previously by Professor Waksman and his team, different batches of the same *Streptomyces* species undergoing the same fermentation process using the same fermentation media does always not produce the same final product yield; the slight variation in production may be explained by the preferential substrate utilization by a specific strain (Waksman et al., 1948). Nevertheless, fermentation process using *Streptomyces* species remains one of the most frequently used methods to manufacture important drugs and their intermediates for medicinal use (Thiry and Cingolani, 2002; Hewitt and Nienow, 2007). Before scaling up the production using large industrial-grade bioreactors, it is crucial to “fine-tune” the media composition used for the production of the compound of interest in lab-scale batch fermentation systems. For instance, Fermentation Medium 3 (FM3) has been optimized previously and used as fermentation media to evaluate bioactive compounds produced by MUSC 137^T^ and MUM 256. As a complex media, FM3 contains nutrients, such as peptone, starch and yeast extract which are essential both for growth and also to support secondary metabolite production in *Streptomyces*. Furthermore, researchers have discovered that fermentation conditions, such as pH, salinity and shaking speed have profound effects on the production of secondary metabolites in *Streptomyces*. As an example, clavulanic acid production in *Streptomyces clavuligerus* was not only affected by media composition (e.g., carbon and nitrogen source), but also by the addition of phosphate in fermentation medium. Phosphate concentration has profound effects on clavulanic acid production with alterations showing a biphasic response, in which low or high concentration of phosphate in media produced low clavulanic acid yield (Ser et al., 2016a). In addition to that, the effect of agitation and/or aeration throughout the fermentation process also affects secondary metabolite production in *Streptomyces* as these bacterial cells might be exposed to oxidative stress and promote the neighboring cells to produce secondary metabolites in an attempt to survive and protect against the challenge (Toma et al., 1991; Joshi et al., 1996; Parekh et al., 2000; Rosa et al., 2005).

## Recovery of bioactive compounds from *Streptomyces* extracts and its screening process

Aside from the optimization of the fermentation process, the choice of solvent and extraction methods are also key to determining the yield and purity of final product. Depending on the applications, different extraction protocols have been used in the bioactivity studies on *Streptomyces* species, ranging from simple extractions using solvents (e.g., methanol, hexane, ethyl acetate) to complicated instrumentations with separation columns like in high performance liquid chromatography (HPLC) (Bóna-Lovász et al., 2013; Ser et al., 2015b; Tan et al., 2015; Özakin et al., 2016). Additionally, factors in selecting extraction techniques also include, but are not limited to, extraction duration, instrument and consumable costs, instrument availability, environmental, safety and health issues (Yan et al., 2008; Henderson et al., 2011; Byrne et al., 2016). For instance, fermentation products of MUSC 137^T^ and MUM 256 were extracted using methanol before being subjected to removal of solvent using a rotary evaporator. The main advantage of using methanol as a solvent is its low boiling point that allows efficient isolation of compounds produced by these streptomycetes without excessive heating (Rudd and Hopwood, 1980; Shimizu et al., 2001; Kumar et al., 2014).

As streptomycetes are known to produce various compounds, different strategies have been deployed to investigate and explore potentially useful bioactive compounds. Typically, there are two main approaches in drug discovery: (a) bottom-up strategy that focuses on seeking compounds/agents that affect molecules that might be critical to disease; and (b) top-down strategy which emphasizes searching for compounds/agents that affect cellular processes that might be critical to disease (Prendergast, 2001; Dias et al., 2012). Regardless of the strategy chosen, streptomycetes derived from mangrove have demonstrated their potential in producing a number of promising bioactive compounds with anticancer and antitumor properties, including macrocyclic lactones, indolocarbazoles, and phenazines (Conda-Sheridan et al., 2010; Fu et al., 2012; Kondratyuk et al., 2012; Yuan et al., 2013). Chemical analysis using chromatographic and/or spectroscopic techniques is often performed as a sequelae of hopeful findings from these bioactivity screening as an attempt to dereplicate and identify potential chemopreventive drugs in these extracts (Ser et al., 2015b, 2016b; Tan et al., 2015). Through gas chromatography-mass spectrometry, a total of 13 compounds were detected from extracts of MUSC 137^T^ and MUM 256; three compounds were found to be produced by the strain including a phenolic compound phenol,2,4-bis(1,1-dimethylethyl)-, and two cyclic dipeptides: pyrrolo[1,2a]pyrazine-1,4-dione, hexahydro- and pyrrolo[1,2a]pyrazine-1,4-dione, hexahydro-3-(phenylmethyl)-. Phenolic compounds have been widely recognized as potent chemopreventive agents, acting as antioxidants and modulators of intracellular signaling processes involved in initiation/promotion of cancer (Ramos, 2008). On the other hand, the cyclic dipeptides family comprises structurally diverse heterocyclic compounds, typically with two amino acid residues and have demonstrated a broad range of biological activities properties including antitumor, antifungal, antibacterial, antioxidant and neuroprotective properties (Giessen and Marahiel, 2015). Given that MUSC 137^T^ and MUM 256 produce compounds with antioxidant and cytotoxic potentials, the understanding of their mechanism of action could potentially be a major step forward in the search of chemopreventive/chemotherapeutic agents.

## Way to go: exploitation of *Streptomyces* for chemoproventive compounds

To date, the etiology of cancer remains somewhat debatable; as a chronic disease, cancer develops when cells become insensitive to chemical signaling molecules that modulate cell growth. This abnormality then leads to uncontrolled cell growth before spreading and invading surrounding tissues and organs. Even though genetics plays a role in cancer initiation and progression (i.e., hereditary/familial cancer), some cancers are associated with environment and lifestyle behaviors, such as smoking, obesity and lack of exercise. The current therapy of cancer usually involves systematic therapy whereby drugs are administered intravenously or orally and/or “local” therapy that may include surgery and radiation therapy. Many currently available anticancer drugs exhibit their anticancer activity by inducing apoptosis of cancer cells (Hickman, 1992; Lee et al., 2007). Programmed-cell death or apoptosis is a complex process involving the affected cells undergoing a self-destruction cascade, and represents an important target for preventive strategies against cancer (Sun et al., 2004; Wenzel et al., 2005). Drugs targeting the apoptotic pathway are categorized into several classes: (a) alkylating agents which bind covalently to DNA and trigger apoptosis; (b) antimetabolites which impede DNA and RNA synthesis; (c) antimicrotubules which prevent completion of mitosis; (d) topoisomerase inhibitors that limit DNA unwinding and synthesis; and (e) cytotoxic antibiotics which can have multiple mechanisms of action. In fact, most of the cytotoxic antibiotics in use are derived from *Streptomyces* species. One of the “classic” drugs derived from *Streptomyces* would be the anthracyclines family which includes doxorubicin and daunorubicin. These drugs can act at multiple levels to promote apoptosis of cancer cells: (i) at DNA synthesis stage by inhibiting DNA and RNA synthesis by intercalating between base pairs of DNA/RNA strand and/or preventing relaxing of supercoiled DNA (i.e., topoisomerase inhibition); (ii) at protein level via generation of reactive free radicals that damage cellular components; or (iii) at both levels through induction of histone eviction from chromatin (i.e., chromatin remodeling) which may eventually cause DNA double strand breaks (Szuławska and Czyz, 2006; Glozak and Seto, 2007; Pang et al., 2015). Interestingly, the fact that histones play an important role in chromatin remodeling has opened up another possibility in the war against cancer. As histone acetylation occurs, the local chromatin transforms, displaying a more relaxed structure which can lead to greater levels of gene transcription; meanwhile, histone deacetylation represses transcription by increasing histone DNA interaction (Jones and Baylin, 2002; Verdin and Ott, 2015). Therefore, the aberrant regulation of histone acetylation has been linked to carcinogenesis as this would contribute to altered transcriptional regulation of genes involved in various important biological processes, such as cell cycle regulation, differentiation, apoptosis, cell adhesion and angiogenesis. Similar to anthracyclines, another group of compounds, cyclic dipeptides can not only trigger apoptosis by modifying histones and inhibiting topoisomerase I activity, but these compounds can also enhance the efficiency of DNA-targeted anticancer drugs (Graz et al., 2000; Marks et al., 2000; Rhee, 2002; Kim et al., 2003; Brauns et al., 2004, 2005). For instance, trichostatin A, isolated from metabolites of *Streptomyces hygroscopicus*, as a histone deacetylase inhibitor was shown to increase the killing efficiency of VP-16, ellipticine, doxorubicin and cisplatin against cancer cells (Kim et al., 2003).

**Figure 3:**
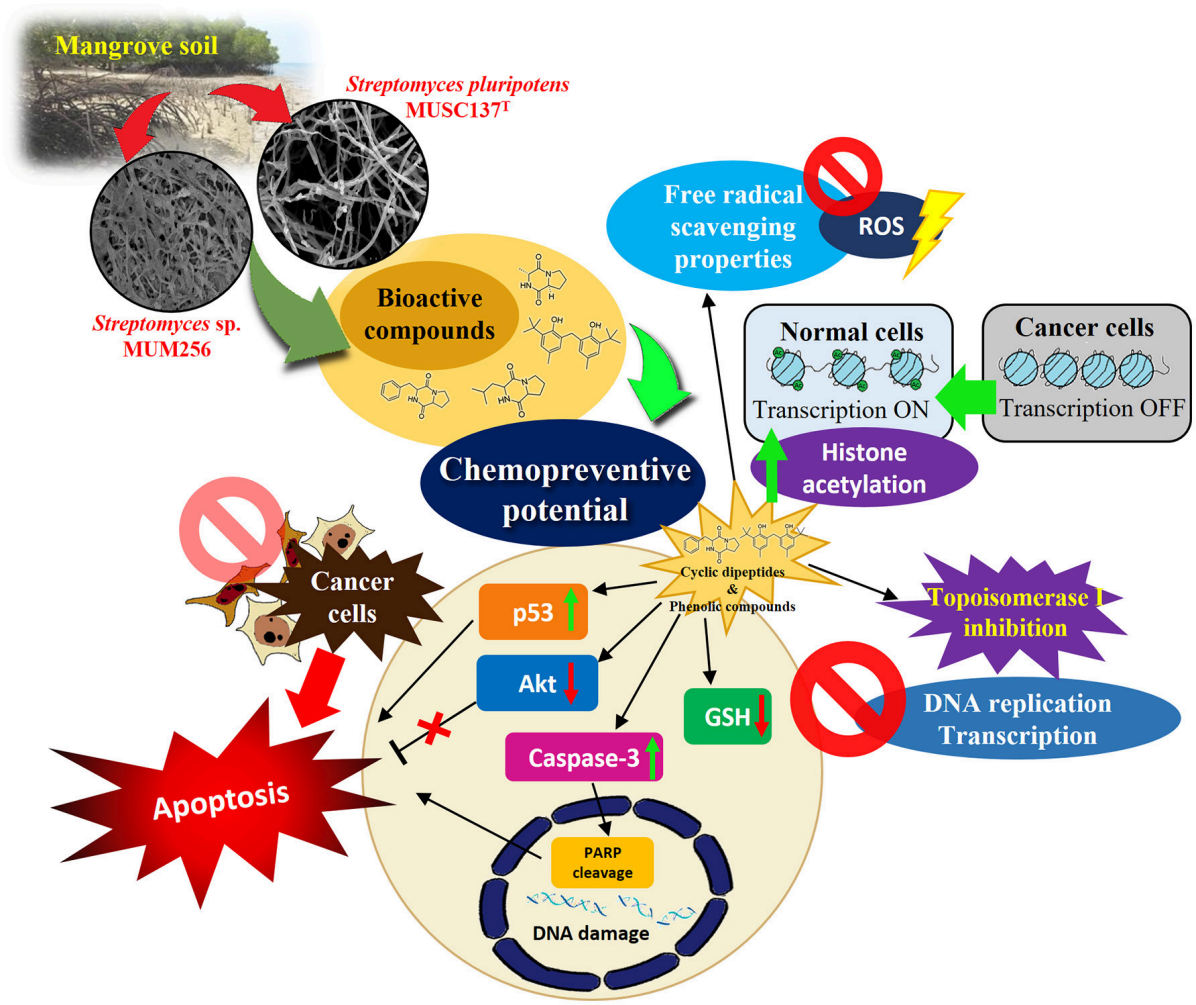
The chemopreventive potentials of bioactive compounds detected in mangrove-derived *Streptomyces pluripotens* MUSC 137^T^ and *Streptomyces* sp. MUM 256 extracts mediated through a wide variety of mechanisms.

In the study of MUSC 137^T^ and MUM 256, there were several interesting cyclic dipeptides that have been detected in other *Streptomyces* species. Previously, our group has detected another novel *Streptomyces* species, MUSC 136^T^ extract containing cyclic peptides that may induce p53 associated apoptotic cell death pathway through the downregulation of intracellular glutathione (GSH) content in colon cancer cells (Ser et al., 2016b). Among the detected cyclic dipeptides in MUSC 137^T^ and MUM 256, pyrrolo[1,2a]pyrazine-1,4-dione, hexahydro-3-(phenylmethyl)- have been featured numerous times in the literature, notably for its growth inhibitory effects on MCF-7, HT-29 and HeLa cancer cell lines (Brauns et al., 2004). The same compound has also produced cytotoxic effect against HT-29 which is found to be mediated through induction of apoptosis. After taking a closer look at its mechanism of action, it was discovered that pyrrolo[1,2a]pyrazine-1,4-dione, hexahydro-3-(phenylmethyl)- not only causes chromatin remodeling, but it also induces PARP cleavage and also increases caspase-3 activity, which eventually triggers apoptosis in HT-29 (Brauns et al., 2005). Meanwhile, another research group has urged for further study on inhibition mechanism of pyrrolo[1,2a]pyrazine-1,4-dione, hexahydro-3-(phenylmethyl)- on DNA topoisomerase I as the compound exhibited more potent inhibition activity than another drug-in-use for cancer treatment, camptothecin (Rhee, 2002). Based on collective evidence, these groups of chemical compounds may potentially account for the antioxidant and cytotoxic properties of the *Streptomyces* sp. MUSC 137^T^ and MUM 256 extracts through a wide variety of mechanisms (Figure 3). The apoptosis-inducing effects of the extracts in the cancer cells may be mediated through the inactivation of Akt1 or activation of p53 signaling pathways that trigger apoptotic signaling cascades. In addition to that, cyclic dipeptides like pyrrolo[1,2a]pyrazine-1,4-dione, hexahydro-3-(phenylmethyl)- may promote cytotoxic effects of MUSC 137^T^ and MUM 256 extracts by the inhibition of DNA topoisomerase I which is important during transcription and DNA replication. In addition to killing and growth inhibitory effects, the extracts of MUSC 137^T^ and MUM 256 may possibly modify histone acetylation status in cancer cells (Graz et al., 2000) which could lead to several biological processes, such as cell cycle arrest, differentiation and apoptosis. Apart from that, antioxidants present in the extracts can induce the cytotoxic effects through modulation of intracellular antioxidant status of cancer cells as evidenced by the down-regulation of intracellular GSH content which subsequently leads to induction of oxidative stress-mediated cell death. As a whole, these findings substantiate the potential of mangrove derived *Streptomyces* sp. such as strain MUSC 137^T^ and MUM 256, as valuable sources of bioactive compounds which merit further investigation for future development of chemopreventive agents.

The importance of isolating, and subsequently mass producing any compound of interest can be illustrated using penicillin as the classic example. Alexander Fleming was the first to observe the “killing” power of the mold, *Penicillium notatum* in 1928 (Bennett and Chung, 2001; Ligon, 2004), and together with his colleagues, they tried numerous methods to extract the active constituent from the “mold juice” but to no avail. It was not till Howard Florey, Ernst Chain and their team at the Oxford University developed a process to extract it that penicillin was transformed from a laboratory curiosity to a “magic bullet.” In order to have enough yield after the difficult purification process using column chromatography, they had to scale up their fermentation process. After all these years, similar techniques are still in use as part of the drug development process. Likewise, there are several possible approaches to increase the production yield of bioactive products from *Streptomyces* species, including but not limited to media optimization. The advancement of **next generation sequencing** (NGS) has opened a new window for researchers to tap into the genomic potential of bacteria including the drug prolific streptomycetes.

KEY CONCEPT 3 **Next generation sequencing**

Along with media optimization, advances in next generation sequencing greatly benefit the drug discovery process in *Streptomyces* sp., particularly in the identification and expression of “silent” or cryptic biosynthetic gene clusters.

The first complete genome of model actinomycete, *Streptomyces coelicolor* A3(2) was available in 2002 (Bentley et al., 2002). The genome of *S. coelicolor* A3(2)—consisting of 8,667,507 bp—is more than two times larger than the genome size of *Bacillus subtilis* (4,214,810 bp) and thus it was published as the largest completely sequenced bacterial genome (Kunst et al., 1997; Bentley et al., 2002). In the same report, the authors also noted the presence of more than 20 gene clusters responsible and/or related to secondary metabolite production; this genomic information further highlighted the important role of *Streptomyces* in drug discovery. Accompanied by substantial improvement in sequencing technology, numerous databases are now available to assist in the genome mining process, particularly for the prediction of biosynthetic gene clusters. As one of the popular tools in identifying the presence of biosynthetic gene clusters, antiSMASH has made significant contributions in microbial natural product research and the web-based system has processed over 260,000 jobs in the past 5 years (Blin et al., 2016). Consequently, this valuable genetic information then allows for a more specific approach than the conventional random mutagenesis method in purposefully directing strain improvements for higher yield of bioactive products that comes with a much reduced cost (Demain and Adrio, 2008; Li and Vederas, 2009; Baltz, 2011). In particular, researchers could tackle the product yield problem via genetic manipulations: (a) modifying expression of regulatory genes; (b) amplification/ duplication of biosynthetic gene clusters; (c) deletion/disruption of competing biosynthetic gene clusters (Baltz, 2011). Conversely, some *Streptomyces* strains have been used previously as hosts to express heterologous secondary metabolite gene clusters including *S. albus* J1074 for the production of macrolide antitumor antibiotics, iso-migrastatin and anthracycline, steffimycin (Gullón et al., 2006; Feng et al., 2009; Baltz, 2010). This alternative method is certainly useful for slow-growing strains and/or those which are not amenable to industrial-scale fermentations. By the same token, heterologous gene expression also successfully “awakened” a number of cryptic/silent biosynthetic gene clusters within *Streptomyces* genome, allowing its full exploitation for bioactive compounds (Reen et al., 2015). In general, *Streptomyces* genomes could essentially represent a “gold mine” for modern drugs that await discovery, as only a fraction of these are expressed during standard fermentations (Zazopoulos et al., 2003; Baltz, 2011). To highlight this point, previous work by Zazopoulous and team discovered that eight out of 50 (16%) previously isolated actinomycetes contained biosynthetic loci containing the enediyne warhead cassette; however, none of these strains was reported to produce enediynes (Zazopoulos et al., 2003). Under these considerations, it has become apparent that genomic mining targeting *Streptomyces* species could unlock the hidden potential of biosynthetic pathways, as well as uncovering pathways which have been previously overlooked. Some studies have indicated the potential biosynthesis difficulties in heterologous hosts due to lack of regulatory, enzymatic or metabolic requirements, Zhang et al. (2017) has recently established an alternative method to activate silent biosynthetic gene clusters in *Streptomyces* genomes—inducing expression of these gene clusters via the CRISPR technology. The CRISPR-Cas9 system -a powerful genome editing technique- is mainly used to delete or knock-out genes (Cobb et al., 2014; Doudna and Charpentier, 2014). However, Zhang et al. (2017) have successfully induced expression of biosynthetic gene clusters in *Streptomyces roseosporus* NRRL15998 by introducing/”knocking-in” a heterologous promoter, kasO^*^ promoter in the upstream region of the gene clusters using CRISPR-Cas9 system. The initial analysis of *S. roseosporus* genome has revealed 29 predicted biosynthetic gene cluster; out of which, one of them showed >90% sequence identity to polycyclic tetramate macrolactam (PTM) gene cluster in *Streptomyces lividans*. As previous work has failed to induce cluster expression in heterologous system, the team decided to employ a knock-in method on the native *S. roseosporus* host. Through this technique, the team successfully activated the expression PTM biosynthetic genes and stimulated production of two PTMs. The continual growth in genome sequencing and mining tools, such as the availability of genome editing technique like CRISPR-Cas9 has truly opened new windows in microbial drug discovery, especially for the identification of novel compounds from *Streptomyces* sp.

Besides exploiting genomic sequence data from culturable bacteria, some researchers have also harnessed the metagenomic approaches in microbial drug discovery. Metagenomic approaches can potentially harness the bioactive potential of environmental bacteria from essentially any environmental source, through the direct cloning of DNA while bypassing the requirement of cultivation (Milshteyn et al., 2014; Trindade et al., 2015). In reality, it has been widely accepted that up to 99.8% of the microbes present in various environments are not readily culturable. There are two main types of screening methods in this culture independent approach: (a) sequence-based methods involving sequencing and bioinformatics analysis of metagenomic samples to identify biosynthetic gene clusters of interest, and (b) functional methods which includes direct functional screening of metagenomic clones for specific activities to identify clones of interest. Nonetheless, many studies have expressed difficulties in partial genome assembly involving metagenomes with high complexity, as these samples normally contain as many as 10^4^-10^5^ unique species (which is frequently observed in soil samples) (Streit and Schmitz, 2004; Milshteyn et al., 2014). As for studies involving *Streptomyces* members, most of the studies have employed a PCR-based, “sequence tag” approach given that most of the biosynthetic diversity originates from a relatively small number of biosynthetic classes (i.e., nonribosomal peptide synthase (NRPS), polyketide synthase (PKS), isoprene, sugar, shikimic acid, alkaloid, ribosomal peptide), in which most of these classes show common, highly similar conserved biosynthetic domains (Lee et al., 2014a,b; Milshteyn et al., 2014). As a matter of fact, the mining of NRPS and PKS genes have allowed discovery of many bioactive streptomycetes from various environments, while revealing the biosynthetic diversity of the environmental habitat (Hakvåg et al., 2008; Lee et al., 2014b; Maciejewska et al., 2016). The availability of genome sequences from metagenomics analysis could speed up the drug discovery process as this technique bypasses the need of isolating and purifying the cultures. Upon retrieving the gene sequences of biosynthetic gene clusters responsible for the production of bioactive compound of interest, researchers can now opt for techniques, such as gene synthesis and heterologous expression to induce synthesis of desired product (Rodríguez-Mata et al., 2013). The progressive development of molecular tools, such as NGS technology and genome editing techniques definitely improve the understanding of *Streptomyces* sp., which is vital for better harnessing their bioactive potential to tackle human chronic diseases and deadly pathogens.

In summary, *Streptomyces* species are truly fascinating microorganisms, producing a variety of bioactive compounds with diverse structures. While numerous drugs have been isolated from terrestrial streptomycetes, researchers have now begun to turn their attention to alternative locations including the mangrove forest in the hunt for novel, bioactive strains. Our group, for example, has isolated numerous strains of novel streptomycetes and other actinobacteria from the mangrove forest in Malaysia (Lee et al., 2014b; Ser et al., 2015a, 2016b; Tan et al., 2015; Azman et al., 2017; Zainal et al., 2016). Apart from antioxidant potential, *S. pluripotens* MUSC 137^T^ and *Streptomyces* sp. MUM 256 are capable of producing bioactive compound(s) that have demonstrated potential to kill cancer cells. Further investigations to elucidate the action mechanisms of these bioactive compounds may hold promise as chemopreventive or chemotherapeutic agents; possibly with the application of advanced genomics and bioinformatics technologies which have revolutionized microbial natural products discovery. With the fast development of sequencing technologies and genome editing techniques, these streptomycetes hold tremendous promise for the development of new anticancer and/or chemopreventive drugs.

## Author contributions

The literature review, data analysis and manuscript writing were performed by HS, LT, and JL, while KC, AD, SS, PP, NA, TK, BG, and LL provided vital guidance, technical support and insight. LL and BG founded the research project.

### Conflict of interest statement

The authors declare that the research was conducted in the absence of any commercial or financial relationships that could be construed as a potential conflict of interest.
