# Supplementary material for: Focused Review: Cytotoxic and Antioxidant Potentials of Mangrove-Derived Streptomyces
Source: Front Microbiol. 2017 Nov 1;8:2065. doi: 10.3389/fmicb.2017.02065 (PMC5672783; doi:10.3389/fmicb.2017.02065)
Supplement: Supplementary file 1 [file Table1.DOCX]

Supplementary Material

Focused Review: Cytotoxic and antioxidant potentials of mangrove-derived *Streptomyces*

**Hooi-Leng Ser^1,2^, Loh Teng-Hern Tan^1^, Jodi Woan-Fei Law^1^, Kok-Gan Chan^3^, Acharaporn Duangjai^4,5^, Surasak Saokaew^1,5,6^, Nurul-Syakima Ab Mutalib^7^, Tahir Mehmood Khan^1,8^, Bey-Hing Goh^1,5^*, Learn-Han Lee^1,5*^**

^1^ Novel Bacteria and Drug Discovery (NBDD) Research Group, School of Pharmacy, Monash University Malaysia, 47500 Bandar Sunway, Selangor Darul Ehsan, Malaysia

^2^ Biomedical Research Laboratory, Jeffrey Cheah School of Medicine and Health Sciences, Monash University Malaysia, 47500 Bandar Sunway, Selangor Darul Ehsan, Malaysia

^3^ Division of Genetics and Molecular Biology, Institute of Biological Sciences, Faculty of Science, University of Malaya, Malaysia

^4^ Division of Physiology, School of Medical Sciences, University of Phayao, Phayao, Thailand

^5^ Center of Health Outcomes Research and Therapeutic Safety (Cohorts), School of Pharmaceutical Sciences, University of Phayao, Phayao, Thailand

^6^ Pharmaceutical Outcomes Research Center (CPOR), Faculty of Pharmaceutical Sciences, Naresuan University, Phitsanulok, Thailand

^7^ UKM Medical Molecular Biology Institute (UMBI), UKM Medical Centre, Universiti Kebangsaan Malaysia, Kuala Lumpur, Malaysia

^8^ Department of Pharmacy, Absyn University Peshawar, Peshawar, Pakistan

*** Correspondence:** Learn-Han Lee, Novel Bacteria and Drug Discovery Research Group, School of Pharmacy, Monash University Malaysia, 47500 Bandar Sunway, Selangor Darul Ehsan, Malaysia. lee.learn.han@monash.edu; leelearnhan@yahoo.com; Bey-Hing Goh, Novel Bacteria and Drug Discovery Research Group, School of Pharmacy, Monash University Malaysia, 47500 Bandar Sunway, Selangor Darul Ehsan, Malaysia. goh.bey.hing@monash.edu

# Supplementary Table

**Supplementary Table 1:** Cultural characteristics of *Streptomyces pluripotens* MUSC 137^T^ and *Streptomyces* sp. MUM 256.

|  | *Streptomyces pluripotens* MUSC 137^T^ | *Streptomyces* sp. MUM 256 |
| --- | --- | --- |
| Growth on ISP 2  (Aerial/substrate mycelium color) | Well  (Yellowish gray/brilliant yellow) | Well  (Light yellow/pale yellow) |
| Growth at:  28 °C  48 °C  pH 6.0  pH 10.0  4% NaCl  10% NaCl | +  +  +  −  +  − | +  −  +  +  +  − |
| Haemolytic activity | − | + |
| Catalase | + | + |
| Hydrolysis activity:  Starch  Carboxymethylcellulose  Casein  Xylan  Chitin  Tributyrin | +  +  −  −  −  − | +  −  −  −  −  − |
| Carbon source utilization:  Dextrin  D-maltose  D-trehalose  D-cellobiose  Gentiobiose  Sucrose  D-turanose  Stachyose  D-raffinose  α-D-lactose  D-melibiose  β-methyl-D-glucoside  D-salicin  N-acetyl-D-glucosamine  N-acetyl-β-D-mannosamine  N-acetyl-D-galactosamine  N-acetyl-neuraminic acid  α-D-glucose  D-mannose  D-fructose  D-galactose  3-methyl glucose  D-fucose  L-fucose  L-rhamnose  Inosine  D-sorbitol  D-mannitol  D-arabitol  Myo-inositol  Glycerol  D-glucose-6-PO_4_  D-fructose-6-PO_4_  D-aspartic acid  D-serine  Gelatin  Glycyl-L-proline  L-alanine  L-arginine  L-aspartic acid  L-glutamic acid  L-histidine  L-pyroglutamic acid  L-serine  Pectin  D-galacturonic acid  L-galactonic acid lactone  D-gluconic acid  D-glucuronic acid  Glucuronamide  Mucic acid  Quinic acid  D-saccharic acid  p-hydroxy-phenylacetic acid  methyl pyruvate  D-lactic acid methyl ester  L-lactic acid  Citric acid  α-keto-glutaric acid  D-malic acid  L-malic acid  Bromo-succinic acid  Tween 40  γ-amino-butyric acid  α-hydroxy-butyric acid  β-hydroxy-D,L-butyric acid  α-keto-butyric acid  Acetoacetic acid  Propionic acid  Acetic acid  Formic acid | -  +  +  +  +  -  +  +  -  -  +  +  +  +  +  +  +  -  +  -  +  +  +  -  +  +  -  -  -  -  +  +  +  +  +  -  -  -  -  +  -  +  -  +  -  -  +  -  -  +  -  -  +  -  -  +  +  +  +  -  -  +  +  +  +  +  -  +  -  +  + | +  +  +  +  +  -  -  -  -  -  -  +  +  +  -  -  +  +  +  +  +  -  -  -  -  +  -  +  +  -  +  +  +  -  -  +  +  +  +  +  +  +  -  +  +  +  +  +  +  +  -  -  -  -  +  +  +  +  +  +  +  +  +  +  +  +  +  +  +  +  + |
| Chemical sensitivity assays  1% sodium lactate  Fusidic acid  D-serine  Troleandomycin  Rifamycin RV  Minocycline  Lincomycin  Guanidine HCl  Niaproof 4  Vancomycin  Tetrazolium violet  Tetrazolium blue  Nalixidic acid  Lithium chloride  Potassium tellurite  Aztreonam  Sodium butyrate  Sodium bromate | +  +  +  +  +  +  +  +  +  +  +  +  +  +  -  +  +  + | +  -  -  -  +  -  -  +  -  -  +  -  +  +  +  +  +  + |
